# Supplementary material for: Filamentous phages reduce bacterial growth in low salinities
Source: R Soc Open Sci. 2019 Dec 11;6(12):191669. doi: 10.1098/rsos.191669 (PMC6936277; doi:10.1098/rsos.191669)
Supplement: Supplementary material [file rsos191669supp1.docx]

**Filamentous phages reduce bacterial growth in low salinities**

Henry Goehlich, Olivia Roth, Carolin C Wendling

**Electronic supplementary material**

**S1: Unsorted consensus matrices**

**Figure S1:** Unsorted consensus matrices per salinity (left: 15 PSU, middle: 11 PSU and right: 7 PSU). Rows represent bacteria and columns represent phages. Black cells indicate infection success in at least 2 out of the three replicates.

**S2: 24h-Growth curves of each strain**

**Figure S2:** 24-hour growth curves per strain and salinity (red: 7 PSU, green: 11 PSU and blue: 15 PSU). Shown are mean and standard-error (n=3). OD was measured at 600nm using an automated plate-reader.

**S3: production of resident phages**

**Figure S3:** Production of resident phages [PFU/CFU] at 15 PSU (left) and 7 PSU (right).

**S4: Bacterial growth rate at 7 relative to 15 PSU**

**Figure S4:** Regression between number of phages causing successful infections in a single *Vibrio* strain at 7 PSU and bacterial growth rate at 7 PSU

**S5: Bacterial growth versus the number of phages causing infections**

**
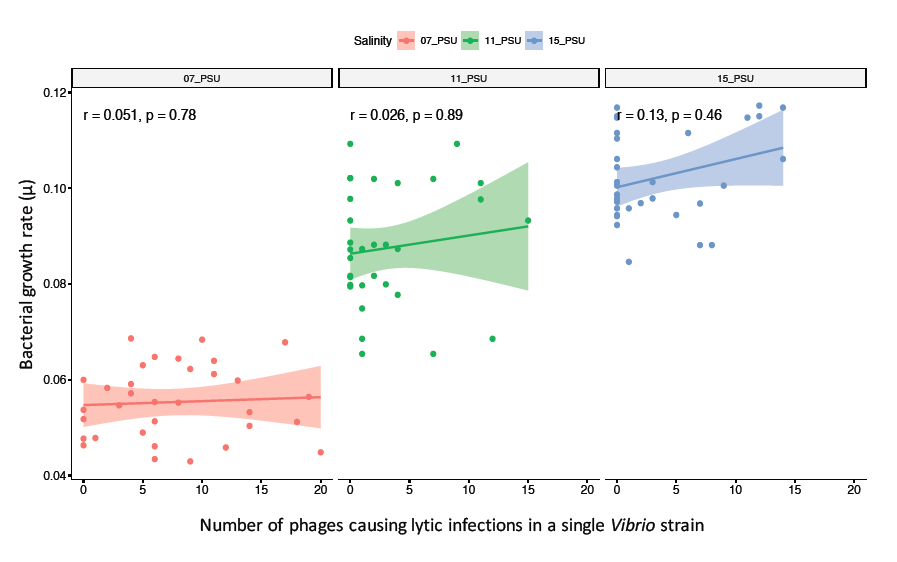
**

**Figure S5:** Regression between number of phages causing successful infections in a single *Vibrio* strain and growth rate of the respective strains at different salinities.
